# A Recognition Method for Rice Plant Diseases and Pests Video Detection Based on Deep Convolutional Neural Network

**DOI:** 10.3390/s20030578

**Published:** 2020-01-21

**Authors:** Dengshan Li, Rujing Wang, Chengjun Xie, Liu Liu, Jie Zhang, Rui Li, Fangyuan Wang, Man Zhou, Wancai Liu

**Affiliations:** 1 Institute of Intelligent Machines, Hefei Institutes of Physical Science, Chinese Academy of Sciences, Hefei 230031, China; dengshan@mail.ustc.edu.cn (D.L.);; 2 Science Island Branch of Graduate School, University of Science and Technology of China, Hefei 230026, China; 3 National Agro-Tech Extension and Service Center, Beijing 100125, China

**Keywords:** rice diseases and pests, deep learning, video detection, deep convolutional neural network, video metrics

## Abstract

Increasing grain production is essential to those areas where food is scarce. Increasing grain production by controlling crop diseases and pests in time should be effective. To construct video detection system for plant diseases and pests, and to build a real-time crop diseases and pests video detection system in the future, a deep learning-based video detection architecture with a custom backbone was proposed for detecting plant diseases and pests in videos. We first transformed the video into still frame, then sent the frame to the still-image detector for detection, and finally synthesized the frames into video. In the still-image detector, we used faster-RCNN as the framework. We used image-training models to detect relatively blurry videos. Additionally, a set of video-based evaluation metrics based on a machine learning classifier was proposed, which reflected the quality of video detection effectively in the experiments. Experiments showed that our system with the custom backbone was more suitable for detection of the untrained rice videos than VGG16, ResNet-50, ResNet-101 backbone system and YOLOv3 with our experimental environment.

## 1. Introduction

The production of crops is of importance, especially to some areas that are particularly lacking in food. The loss of grain caused by crop diseases and pests accounts for more than 10% of the total crop production loss [1]. Therefore, timely detection of those may have a positive effect on increasing crop production. Trying to build a real-time video detection system may be advantageous for the prevention of crop diseases and pests, and it is the purpose of building the rice plant video detection system in the paper.

**Figure 1:**
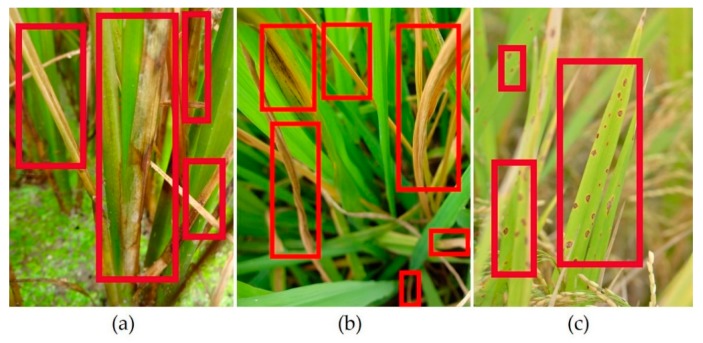
As shown in the red boxes, the lesion spots of rice sheath blight, rice stem borer symptoms, rice brown spot are visualized: (**a**) rice sheath blight, (**b**) rice stem borer symptoms, (**c**) rice brown spot. The images are from our image dataset, which were collected in Anhui, Jiangxi and Hunan Province, China, between June and August, 2018.

Rice is the staple food in China. It may be affected by several diseases and insects, such as rice sheath blight, rice stem borer, rice brown spot. The lesion spot of rice sheath blight is cloud-like, grayish-brown in the middle, and the leaves are withered and yellow, which is caused by fungal infection [2], the pathogen is Rhizoctonia solani Kühn. Rice stem borer symptoms is caused by a kind of insect, Chilo suppressalis. Its symptoms are withered stems and leaves [3]. Rice brown spot is also caused by fungal infection too, the pathogen is Bipolaris oryzae (Breda de Haan) Shoem. Its symptoms are brown spots on leaves and leaf sheaths [4]. The above three kinds of lesions are shown in Figure 1.

Diseases of rice plants could be affected by different factors, such as climatic conditions, lighting conditions, humidity, nutrients, fertilizer, water management, and farming conditions. Manual identification and detection of those diseases are often time-consuming, and the recognition accuracy may not be high. It may result in incorrect diagnosis and misuse of pesticides. Compared with the traditional image recognition methods, such as histograms of oriented gradients (HOG) [5] and scale-invariant feature transform (SIFT) [6], etc., deep learning based methods should have higher accuracy than the human eye [7] and may outperform traditional methods in detecting crop diseases and pests.

With the proposal of convolutional neural networks (CNN) [8], region-based convolutional neural network (R-CNN) [9], Fast-RCNN [10] and Faster-RCNN [11], image object detection has been developed. CNN is one of the most effective methods of image processing [12,13,14,15]. CNN uses a convolutional method to extract image features, which achieves high-level fusion of semantics and deep extraction of features through multi-layer networks [16]. Compared with R-CNN and Fast-RCNN, as using Region Proposal Network (RPN) to extract candidate boxes (i.e., region proposals in the literature [11]), Faster-RCNN improves the detection speed and accuracy [17,18]. VGG net [19] and Residual net [20] have got a high ranking in ImageNet competition.

The video object detection has also been developed. YOLO [21] has obtained real-time performance in video detection, and the detection accuracy of YOLOv3 [22] is higher than YOLO and YOLO9000 [23]. YOLO performs prominently in video object detection. Karpathy et al. present a fusion of CNN models [24], which achieves video content classification on a large scale. Non-maximum Suppression (NMS) is a method to filter out local non-maximum bounding boxes [25]. Han et al. propose a method of “sequence NMS” to select high NMS level boxes from adjacent frames [26]. Galteri et al. use a closed-loop architecture to feedback the detection results to the previous proposals, which makes the bounding box location more accurate [27]. Kang et al. adopt tubelets with convolutional neural network (T-CNN) for video object detection [28].

**Figure 2:**
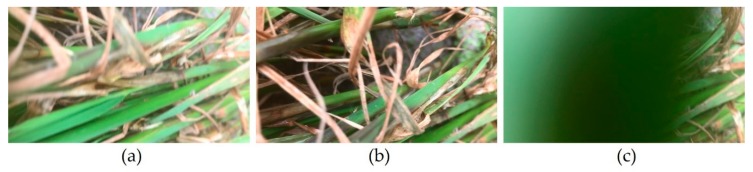
The challenges of rice video detection. Subgraphs (**a**,**b**,**c**) are frames of our video dataset (Video 1 illuminated in Section 2.1). (**a**) Video defocus, (**b**) motion blur, (**c**) part occlusion. Video 1 was captured in Hunan Province, China, July 2018.

**Figure 3:**
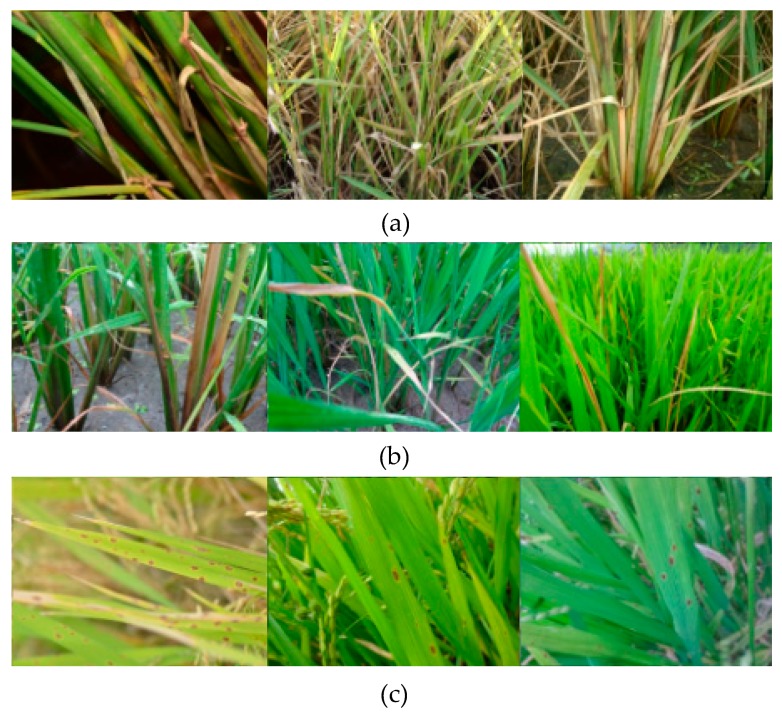
Images of the three kinds of rice diseases and pests. (**a**) Rice sheath blight, (**b**) rice stem borer symptoms, (**c**) rice brown spot. These images were collected in Anhui Province, etc., China, between June and August, 2018.

Despite the success of the above methods, video detection is different from still-image detection in many ways, and often remains challenges such as video defocus, motion blur and part occlusion [29]. The video frames of rice diseases and pests symptoms which contain video defocus, motion blur and part occlusion are shown in Figure 2. Changes of depth of field often lead to video defocus; swift motion, such as leaf motion caused by wind, often leads to motion blur; because of the change of the camera angle, the leaves in the foreground may occlude the lesion spots in the background, which leads to part occlusion. As shown in Figure 3, our images of rice diseases and pests symptoms are clear and with no occlusion, therefore, using the still images to detect rice lesion videos often remains challenges too.

Deep learning methods have been used in the recognition and detection of crop diseases [1,30,31,32,33]. Bai et al. cluster features with neighborhood grayscale method to extract the disease area of cucumber leaves [34], Dorj et al. estimate the fruits of the citrus trees using image processing methods [35], Ma et al. extract the key frames of the video by mean pixels value method [36]. Additionally, many recognition and detection methods have attempted in feature extraction and image segmentation of crop diseases by deep learning [37,38,39].

CNN can be trained to learn the features of images, such as pattern, color, and texture [40]. The plant lesions have these features, which makes CNN suitable for symptomatic detection. Therefore, CNN is an effective method for detecting crop diseases [41,42]. However, multiple diseases in one crop image may reduce the accuracy of disease diagnosis [43]. This may be due to the mutual interference of features between different kinds of lesion spots.

Detection of crop diseases and pests symptoms is always facing difficulties, since the shape of lesion spots is always irregular, and the boundary between spots and surrounding healthy areas is always not obvious. Especially for video detection, the lesion spots would be blurrier in the moving status, and the irregular shape of the spots changes with the change of the camera angle.

In this paper, we develop a video detection system for detecting rice diseases and pests symptoms. The proposed system tries the first step of real-time crop lesion video detection. The system contains a frame extraction module, a still-image detector and a video synthesis module. We use still images from our dataset (Figure 3) to train a still-image neural network model, and use the model to detect video frames (Figure 2). Our system extracts frames from video, then sends the frame to the still-image detector for detection, and synthesizes the detected frame into video. Extraction, detection, and synthesis are performed nearly synchronously. The system can detect multiple kinds of lesion spots in one video. The system is depicted in Section 2.3. We use Faster-RCNN as the framework in the still-image detector. The framework is depicted in Section 2.3.2. A custom deep convolutional neural network (DCNN) backbone of Faster-RCNN is designed in this paper. The backbone is depicted in Section 2.3.3. The DCNN is the first stage of Faster-RCNN, which is used to extract features. The second stage of Faster-RCNN is the localization and classification part, which locates the lesion spots and classifies them. The backbone of Faster-RCNN could be replaced with other structures, such as VGG16, ResNet-50, ResNet-101, and we compare the backbones and the custom DCNN on rice diseases and pests video detection in Section 4.5.

To summarize, we make the following contributions in this paper: (1) we try to propose a set of video detection metrics, which is based on machine learning classifier; (2) we propose a custom DCNN backbone, the backbone is used as the first stage of the “two-stage” detector—Faster-RCNN; (3) we build a video detection system for rice diseases and pests.

## 2. Materials and Methods

### 2.1. Image and Video Datasets

The dataset was composed of images and videos of rice disease and pest lesions, which were collected in Anhui, Jiangxi and Hunan Province, China, between June and August, 2018. The dataset was about rice sheath blight, rice stem borer symptoms, and rice brown spot. These images were taken by mobile phones, such as iPhone7 and HUAWEI P10, and Sony WIFI-controlled camera, whose model type was DSC-QX10. The image resolution of rice sheath blight was 4928 × 3264 pixels as same as the rice brown spot, and the image resolution of rice stem borer symptoms was 2592 × 1944 pixels. The videos were taken only by iPhone7 with 1920 × 1080 pixels per frame, since iPhone often has a clearer video capture. Image and video collection focused on occurrences of heavy infestation.

Training data and test data were composed of 1800 images, 1760 images, and 1760 images for rice sheath blight, rice stem borer, and rice brown spot, respectively (Table 1). 15, 23, and 13 videos of the aforementioned diseases were only used for detection. The videos were not used when training the model. The images were randomly divided into a training set and test set with the ratio of 9:1 before training. As shown in Table 2, Videos 1–3 were selected for detection in this paper, since they contained the presence of multiple disease and pest categories. Video 4 and Video 5 were used for validation. The main symptoms of Videos 1–3 were rice sheath blight, rice stem borer symptoms and rice brown spot, respectively, and the main symptoms of Video 4 and Video 5 were rice sheath blight and rice stem borer symptoms. Video 1 was used for the comparison experiments in Section 4.4, Section 4.5 and Section 4.6. The flow of our system was to train a model with relatively clearer images (as shown in Figure 3), and then use the model to detect relatively blurrier videos frame by frame. The video frames are shown in Figure 2.

**Figure 4:**
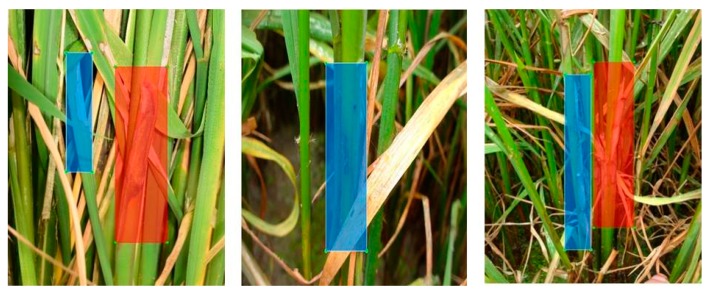
The annotation of rice sheath blight in our datasets. Only lesion spots were annotated, and no withered leaves were annotated. Different colors only denote different annotation boxes, the lesions in the figure are all rice sheath blight. The images were captured in Anhui Province, China, June 2018.

The annotation of rice sheath blight is shown in Figure 4. We did not annotate the subordinate symptoms like withered leaves. We only annotated the lesion spots which were the heavy infestation. These images were used for training and testing the still-image model. It could be seen that the images used for training were clearer compared with video frames shown in Figure 2. Our system was to use the model trained with clear images to detect relatively blurry videos frame by frame, which would be a difficult point for the rice video detection.

### 2.2. Video Detection Metrics

We try to propose a set of evaluation metrics of rice video detection by referring to the image detection metrics [44]. The evaluation metrics are defined as follows:

(1) $$\mathrm{Video}\;\mathrm{precision}=\frac{\mathrm{number}\;\mathrm{of}\;\mathrm{true}\;\mathrm{spots}\;\mathrm{detected}}{\mathrm{number}\;\mathrm{of}\;\mathrm{spots}\;\mathrm{detected}}$$

(2) $$\mathrm{Video}\;\mathrm{recall}=\frac{\mathrm{number}\;\mathrm{of}\;\mathrm{true}\;\mathrm{spots}\;\mathrm{detected}}{\mathrm{number}\;\mathrm{of}\;\mathrm{true}\;\mathrm{spots}}$$

(3) $$F1\;\mathrm{score}=\frac{2\;\times \;\mathrm{video}\;\mathrm{precision}\;\times \;\mathrm{video}\;\mathrm{recall}}{\mathrm{video}\;\mathrm{precision}\;+\;\mathrm{video}\;\mathrm{recall}}$$

“Number of true spots detected” in Equation (1) denotes correctly boxed lesion spots. “Number of spots detected” in Equation (1) includes the number of correctly boxed lesion spots and wrong boxed spots. “Number of true spots” in Equation (2) include the boxed and unboxed ones. The same spot only needs to be counted once in a video.

To simplify the calculation, we only count whether the lesion spots are detected or not. We use the concept of still-image metrics, such as true positive, false positive, true negative, e.g., “number of true spots detected” has the meaning of true positive, as it denotes the correctly boxed lesion spots; “number of spots detected” has the meaning of true positive and false positive, as it denotes correctly boxed lesion spots and wrong boxed spots; “number of true spots” has the meaning of true positive and true negative, as it denotes the boxed and un-boxed lesion spots.

The number of spots in our metrics is counted manually at present as the videos are not annotated. To calculate the metrics automatically needs to annotate the videos [45] manually. Additionally, when using a computer to calculate the metrics, how to judge whether the spots of different frames are the same spot that has been counted is considerably difficult. To calculate them automatically is our next research direction.

Video precision measures how many boxed lesion spots are correct. Video recall measures how many correct lesion spots are boxed out. F1 score is a harmonic average of precision and recall, and comprehensively measures precision and recall. The value of the above equations is always less than 100%, because the denominators are always larger than the numerators according to the definition.

The literature [28] uses the still-image detection metrics to evaluate its video detection, such as the mAP value of frames. We consider the metrics in this paper could better measure video detection, as they are from the standpoint of video, rather than the still frames. For instance, the same lesion may appear in multiple adjacent frames, using still-image metrics of frame may lead to the repeated calculation, which may lead to the statistical deviation, e.g., high precision of a kind of lesion spot may be the repeated statistics of the same lesion in different frames.

The equations above could be extended to multiple categories of spots, i.e., the number of spots in the equations could be replaced by more categories of spots. The video metrics could be extended to “total videos” or “average metrics”. Total video detection metrics are the sum counting of spots of multiple videos. Average video metrics are the sum of the total metrics divided by the number of the corresponding indices.

### 2.3. The Video Detection System

The structure of our video detection system consists of three parts: (1) frame extraction module which can be set to any time interval; (2) still-image detector, with a custom DCNN as the backbone. The DCNN backbone is composed of 12 convolutional layers, and the dimensions of each convolutional layer is specific; (3) video synthesis module. Generating a frame, detecting a frame and synthesizing a frame to the video are synchronous. The detailed algorithm of the video detection system is shown in the Supplementary Materials. The workflow of this video detection system was as follows: we first trained a still-image model with the images from our image dataset, then input a video into the system, the final output of the video detection system was a detected video. Our system was using a model trained with still images (Figure 3) to detect relatively blurry video frames (Figure 2).

#### 2.3.1. Frame Extraction Module

At the beginning of our video detection system, frames are extracted from the input video. Then, the frame just generated is sent to the still-image detector. We extracted every frame in chronological order. The speed was 30 frames per second. The resolution of the generated frame is the same as that of the original video. For the detection of plant lesion spots, reducing the resolution of the image to improve the detection speed, may not be a better choice, because of the irregular shape of the lesion spots and the unclear boundaries of the spots. If reducing the resolution too much, the spots may be much blurrier and harder to detect.

#### 2.3.2. Still-Image Object Detector

**Figure 5:**
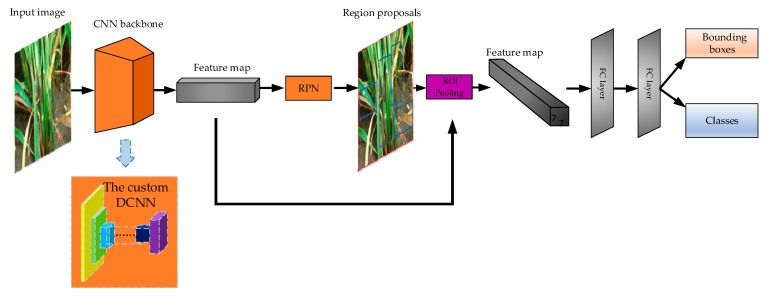
The architecture of the still-image detector, which is using the framework of Faster-RCNN, where the “CNN backbone” is the proposed deep convolutional neural network (DCNN).

The still-image object detector is adapted from Faster-RCNN [11] framework, which is a “two-stage” object detector. In the framework, the first stage can be replaced with other CNNs, such as VGG16, ResNet-50 and ResNet-101, which is the backbone. We designed a custom DCNN architecture as the backbone. The illustration of the backbone is in Section 2.3.3. Our framework is still an end-to-end detection network as the original Faster-RCNN. The architecture is visualized in Figure 5. The following is a detailed description of the framework and each part.

(1) The Faster-RCNN Framework

Faster-RCNN is the representative structure of two-stage object detector. Regions of Interest (RoI) are generated by Region Proposal Network (RPN), and then transferred to the next part for classification and bounding box regression. As shown in Figure 5, the framework includes feature extractor (called backbone in some literature), RPN, RoI pooling part, two full connected (FC) layers.

(2) The Custom DCNN Backbone

Backbone is the first stage of the “two-stage” detector. Backbone’s function is to extract features of the image and generate the feature map. A custom DCNN was designed as the backbone. Feature extractor is important to two-stage deep learning detector, because the quality of feature extraction affects the quality of detection. The architecture of the backbone is illustrated in Section 2.3.3.

(3) RPN

RPN in Faster-RCNN is an alternative to “selective search” in Fast-RCNN. Selective search is to divide the whole picture into many small regions, then perform the neural network which generates the bounding boxes in each small region, and finally output small regions and the bounding boxes. Thus, the efficiency is low. RPN runs the neural network once to extract RoIs, which improves the efficiency.

(4) RoI Pooling Part

RoI pooling part converts region proposals generated by RPN into the feature map of fixed height and width (7 × 7). The advantage of this method is that the size of the input image may not be fixed, as any size of the previous feature map will be RoI pooling into H × 7 × 7 feature map, where H is the number of RoIs [11].

(5) Head of the Faster-RCNN Framework

The head is composed of the last two FC layers, whose function is to combine all the local features to the whole features of the object. The dimension of the two FC layers is 1024 respectively. The FC layer outputs two branches, which are the classification branch and the bounding box branch. Classification branch has four dimensions: three dimensions denoting the three categories of rice lesion spots, and one dimension of the background. We used the SoftMax function to calculate the probability. Bounding box branch has 16 dimensions, which are the product of the four dimensions and the four coordinates of each box. The four coordinates are: top left, top right, bottom left and bottom right.

#### 2.3.3. The Custom DCNN Backbone

For use in the Faster-RCNN framework, we designed a neural network backbone comprised of 12 convolutional layers. The structure of this neural network is depicted in Table 3, where the column “repeat” represents three consecutive repetitions of the corresponding part. The custom network was inspired by the structure of VGG16 [19] and AlexNet [15], and used the same principle of constructing CNN [46], i.e., CNN is composed of input layer, convolutional layer, max-pooling layer and classification layer. We used the max-pooling layer after the convolutional layer, which was verified by Scherer et al. that max-pooling could result in faster convergence and could select out superior features [47]. A deeper network can extract more advanced semantics [48], but it also means the loss of information. On the other hand, the deep network can eliminate useless information which does not contain the features. Thus, we chose the architecture of 12 convolutional layers, which we considered the depth was moderate for video detection. The DCNN backbone achieved a good detection effect compared with VGG16, ResNet-50 and ResNet-101 backbone in our experiments. Section 4.5 compares the video detection metrics of VGG16, ResNet-50, ResNet-101 and our backbone in detail.

The DCNN backbone architecture is made up of four blocks. The channel of the input layer “Image” is 3 because of the red, green, blue channel of an image. The first block consists of five parts: three convolutional layers which have 64 filters with the size of 3 × 3; a local response normalization (LRN) layer; a max-pooling layer with the kernel of 2 × 2, which is used to downsample the data. The second block also includes five parts: three convolutional layers that have 128 filters with the size of 3 × 3; the same LRN layer; the same max-pooling layer. The third block also consists of five parts: three convolutional layers which have 256 filters with the size of 3 × 3, and other layers are the same as the first block. Finally, the last block also consists of five parts, except for the three convolutional layers which have 512 filters with the size of 3 × 3, the rest are also the same as the first block.

We use three convolutional layers in each block, and the convolution kernel of each convolutional layer is 3 × 3, which ensures that the receptive field at the end of each block is large enough, which should be useful for detecting large objects, such as large lesion spots. Meanwhile, as there are only three convolutional layers, and only one max-pooling layer in each block, the information loss caused by convolution and pooling would be reduced to a lower level. The 12 convolutional layer architecture may have high semantic information, which may be sufficient to recognize blurry objects, such as blurry lesion spots.

The novelty of the proposed DCNN are as follows: (1) We changed the order of ReLU layers and put the ReLU layer between two convolutional layers, this might be beneficial for the convergence of the deep network. (2) We set the number of convolutional layers to 12, and the depth might be moderate for high semantic recognition. (3) We set the number of blocks (called “stages” in literature [49]) to 4, and set one pooling layer at the end of each block, which might reduce the loss of input information. (4) We set three adjacent convolutional layers in each block, intended to make the DCNN have a large receptive field. (5) We changed the dimensions of each convolutional layer, which is the “output channels” in Table 3, that we considered this might be beneficial for high semantic recognition too.

Krizhevsky et al. propose local response normalization (LRN) [15]. LRN is integrated into the four blocks to form the custom DCNN backbone. LRN is defined as:

(4) $$B_{x,y}^{i}=\frac{A_{x,y}^{i}}{{\left(k+\alpha \sum_{j=\max\left(0,i-n/2\right)}^{\min\left(N-1,i+n/2\right)}{\left(A_{x,y}^{j}\right)}^{2}\right)}^{\beta }},$$

where $A_{x,y}^{i}$ and $A_{x,y}^{j}$ are the output value of the activation function ReLU, x and y denote the position of the feature map, i and j denote the kernel of the layer, N is the total number of the kernel, n, k, α, and β are the hyper-parameters which are validated in experiments. We used k = 2, n = 5, α = 0.0005, and β = 0.75 in the experiments. LRN’s function is to implement the lateral inhibition inspired by some subordinate neurons [15].

In the process of studying the proposed DCNN backbone, we tried other different architectures, such as increasing or decreasing the total convolutional layers, increasing or reducing the number of the adjacent convolutional layers. Detailed discussion is in Section 4.4.

#### 2.3.4. Video Synthesis Module

The frames detected in the still-image detector contain detection information, such as classes and the detection confidence. Different box colors were used to distinguish different classes in the experiments. Our video synthesis module receives the just detected frame from the still-image detector and synthesizes the frame to video. The resolution of the generated video is the same as that of the frame.

## 3. Experiments

### 3.1. Training

A Linux server with Ubuntu 16.04 was used in the experiments. The server included an Intel i7-6700 K CPU, 64 GB DDR4 RAM, and two NVIDIA Titan X GPUs, with the deep learning platform of Detectron [50]. Before training, we divided the images into a training set and test set with a ratio of 9:1 randomly. Data augmentation [51] such as image flipping and cropping was used in training by the platform Detectron automatically. Pre-trained models could be used in training by Detectron.

The system with the custom DCNN backbone was trained with CUDA 9.1 and cuDNN 7.1.3. Stochastic Gradient Descent (SGD) with momentum was used to converge the model. The learning rate was set to 0.002, and then decreased every 20,000 iterations by a factor of 0.1. The momentum was set to 0.9 with a weight decay of 0.0001. The mini-batch was set to 1. Detectron resizes the input images to avoid GPU memory overflow during training, thus, the maximum size of the input training images was limited to 2500 pixels, while the minimum was limited to 1500 pixels. The iterations were set to 60,000. In the contrastive experiments, the parameters were set to the appropriate values. In Section 4.4, learning rate of 11 convolutional layer DCNN was set to 0.0001, and iterations were set to 80,000; in Section 4.5, learning rate of VGG16 with pre-trained model was set to 0.00025, and the max iteration was set to 100,000; in Section 4.6, since the number of training pictures was 5320, we set the number of iterations to 50,000, and saved the models of 7000, 8000, 9000, 10,000, 20,000, 30,000, 40,000 and 50,000 iterations. The learning rate of YOLOv3 was set to 0.001, which decreased by a factor of 0.1 per 10,000 iterations so that the early iteration models could be tested in our experiments. The above network training was stopped at each max iteration when the resultant loss reached a minimum, which was verified by our experiments.

### 3.2. Setting of the Detection Threshold

The detection threshold of confidence was set to an appropriate value to filter out the detected lesion spots whose detection confidence was lower than this threshold value. Thus, the higher the threshold was set, the fewer lesion spots would be detected.

From the experiments, we learned that, when the video detection threshold was set below 0.2, most of the spots could be detected; when the threshold was set to 0.7, only a few spots could be detected. In order to achieve better detection results, we set the video detection threshold to 0.2, and most of the spots could be detected, with a few redundant overlapping boxes. In our experiments, the three other backbones and YOLOv3 were all set to 0.2.

## 4. Results and Discussion

### 4.1. Results of the Rice Video Detection

**Figure 6:**
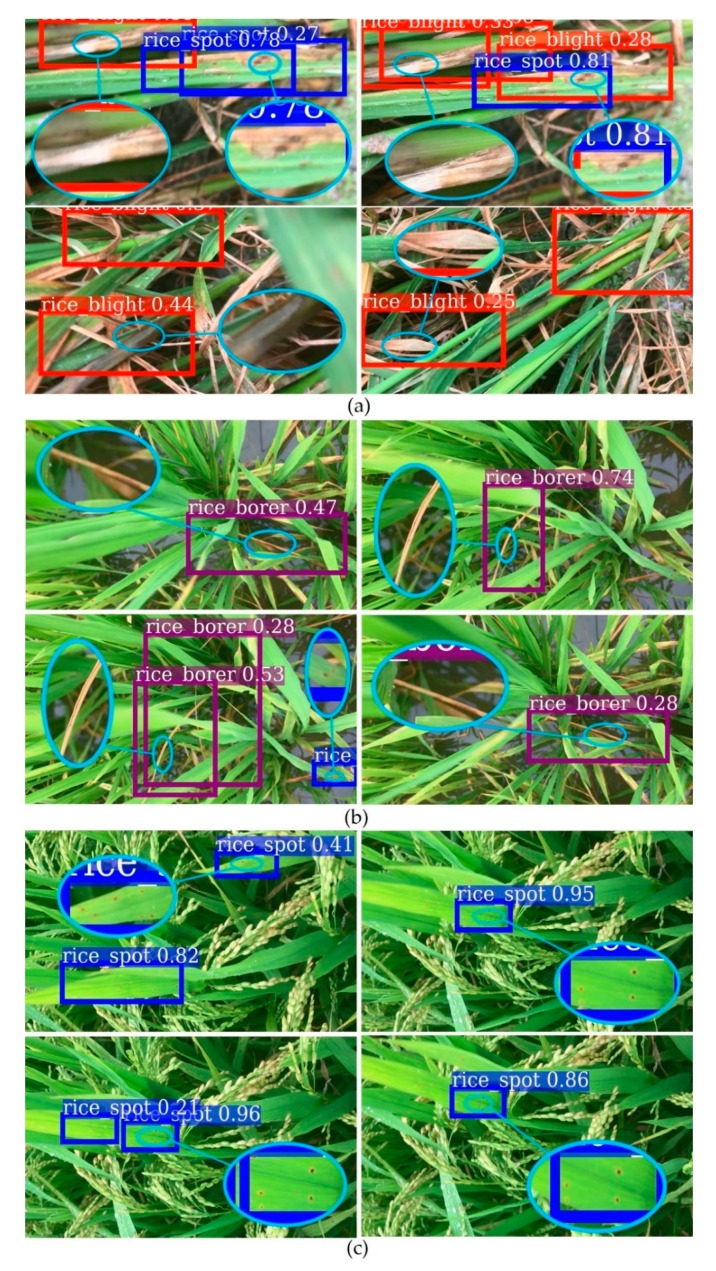
The custom DCNN video detection system results, (**a**) Video 1, which mainly has rice sheath blight. (**b**) Video 2, which mainly has rice stem borer symptoms. (**c**) Video 3, which mainly has rice brown spot. Each video has three categories of rice disease and pest symptoms. Red, purple and blue boxes denote rice sheath blight, rice stem borer symptoms, and rice brown spot respectively. Indigo ellipses magnify the lesion spots, which is realized by Adobe Photoshop CS6. The same kind of lesions is magnified once in each subgraph. Videos 1–3 were not used in the model training. Video 1 and Video 3 were captured in Hunan Province, China, July 2018, Video 2 was captured in Jiangxi Province, China, July 2018.

Figure 6 is the video detection results. Red boxes denote rice sheath blight, purple boxes denote rice stem borer symptoms, and blue boxes denote rice brown spot. Some of the very blurry lesion spots in the videos were not detected. The three categories of rice diseases and pests symptoms could be detected simultaneously. The overlapping boxes may be the reflection of different lesions in Figure 6a. Since the detection threshold was set low, it might also cause the overlapping boxes. The detection speed was about 0.1 s per frame.

Table 4 shows the lesion spots statistics of Videos 1–3. “Number of true spots”, “number of spots detected” and “number of true spots detected” are illustrated in Section 2.2. The way these lesions were counted is, a spot hooped by multiple boxes was only counted once. Multiple lesion spots hooped by a large box were counted all. The proposed video detection metrics of Videos 1–3 are listed in Table 4, too.

In Table 4, the results of video recall and video precision are calculated by Equation (1) and Equation (2). For instance, video recall of Video 1 is the quotient of “number of true spots detected” divided by ”number of true spots”, therefore, the value is 68.0%, as a result of 34 divided by 50.

### 4.2. Confusion Matrix

**Figure 7:**
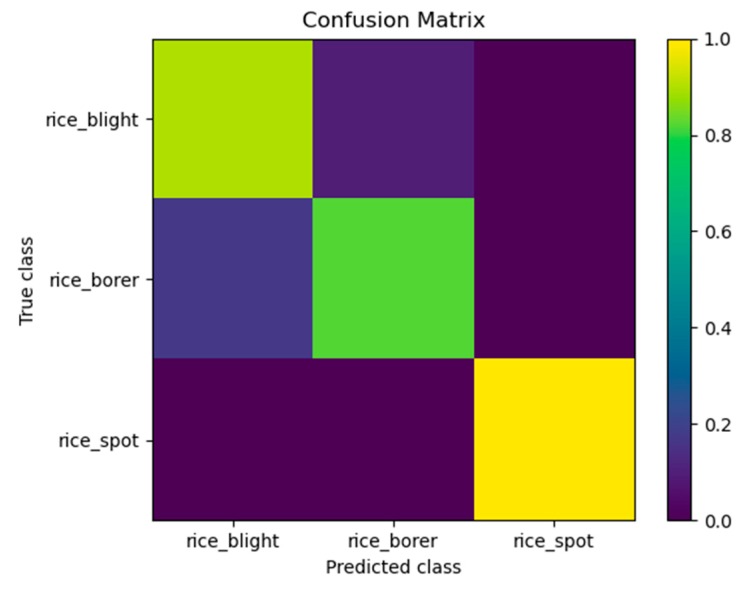
Confusion matrix of Video 1 which contains rice sheath blight, rice stem borer symptoms and rice brown spot. Video 1 was captured in Hunan Province, China, July 2018, which was illustrated in the caption of Figure 6. The input data of the confusion matrix was counted manually from Video 1.

The detector would be confused about the three categories of rice diseases and pests. Since we used image-training models to detect videos, the property of video defocus, motion blur and part occlusion of videos, and the complex background of videos were all the factors of the confusion. We presented a confusion matrix of the video detection results of Video 1 in Figure 7. From Figure 7, the performance of the detector could be visualized by different colors of the figure. The numbers of the true spots of rice sheath blight, rice stem borer symptoms and rice brown spot were 27, 11 and 12 respectively. Three spots of rice sheath blight were misclassified as rice stem borer symptoms. Three spots of rice stem borer symptoms were misclassified as rice sheath blight. Rice brown spot was classified correctly. The numbers above were all counted manually.

### 4.3. Analysis and Validation of the Results

We analyze that the reasons why some lesion spots in the videos are not detected, or wrong detected, and the detection confidence is not very high is that the spots in the videos are relatively blurry and distorted compared with the still-images which are used to train the model, which leads to the features may be different from the still-image ones from our dataset. The annotated lesion spots of the sample images we used for training the still-image models are regular, standard and clear, while spots in videos may be distorted, obscured, and blurry. There may be other reasons, like the difficulties in rice video detection illustrated in Section 4.8.

**Figure 8:**
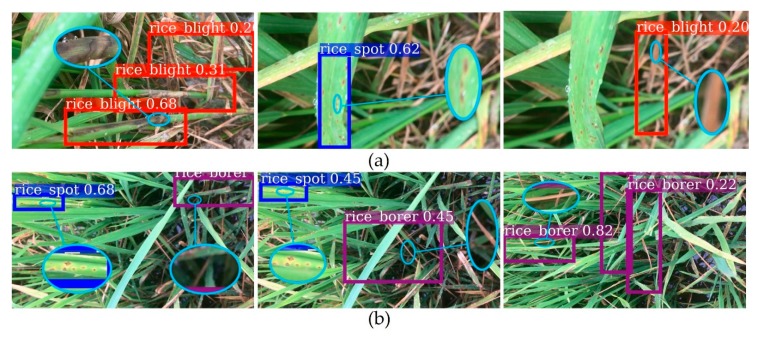
The custom DCNN video detection system qualitative results of Videos 4 and 5. (**a**) Video 4, which mainly has rice sheath blight. (**b**) Video 5, which mainly has rice stem borer symptoms. Red, purple and blue boxes denote rice sheath blight, rice stem borer symptoms, and rice brown spot respectively, and indigo ellipses magnify the lesion spots, which is realized by Adobe Photoshop CS6. The same kind of lesions is magnified once in each subgraph. Video 4 and Video 5 did not participate in model training. Video 4 was captured in Hunan Province, China, July 2018, Video 5 was captured in Jiangxi Province, China, July 2018.

One of the difficulties of our system was that we used clear photos to train a model, and then used the model to detect a relatively blurrier video frame by frame. We could see the detection effect in Figure 6. The system needed to be further validated. We used Videos 4 and 5 to do the validation experiments, for verifying the detection effect of rice sheath blight and rice stem borer symptoms, which had similar symptoms of withered stem and leaves. The qualitative results are visualized in Figure 8, and metrics are listed in Table 5. Our custom DCNN video detection system achieved good detection results on Videos 4 and 5.

### 4.4. Study on Different Depth Architecture of the Proposed DCNN Backbone

We used Video 1 to test and analyze. Each contrastive experiment of different architecture was set with the appropriate hyperparameter after parameter adjustment experiments, the parameters were illustrated in Section 3.1.

Table 6 is our experiment results. In the third line, the “custom DCNN” is the final architecture we adopt. We tested backbone architecture with 14 convolutional layers and 11 convolutional layers respectively. the metrics of 14 and 11 convolutional layers were lower than the ultimate DCNN.

### 4.5. Comparison with Other Backbones

We selected VGG16, ResNet-50, ResNet-101 and our custom DCNN as backbones of the still-image detector (Faster-RCNN framework), to evaluate the performance of these backbones in rice video detection. We used the same image dataset to train models of these backbones, and used Video 1 for detection. The same experimental environment such as the Linux server, GPU, CUDA, and cuDNN was selected. The three other backbones achieved their best video detection effects after the hyper-parameter adjustment. With our experimental environment and dataset, the custom DCNN backbone achieved good experimental results in the rice video detection.

**Figure 9:**
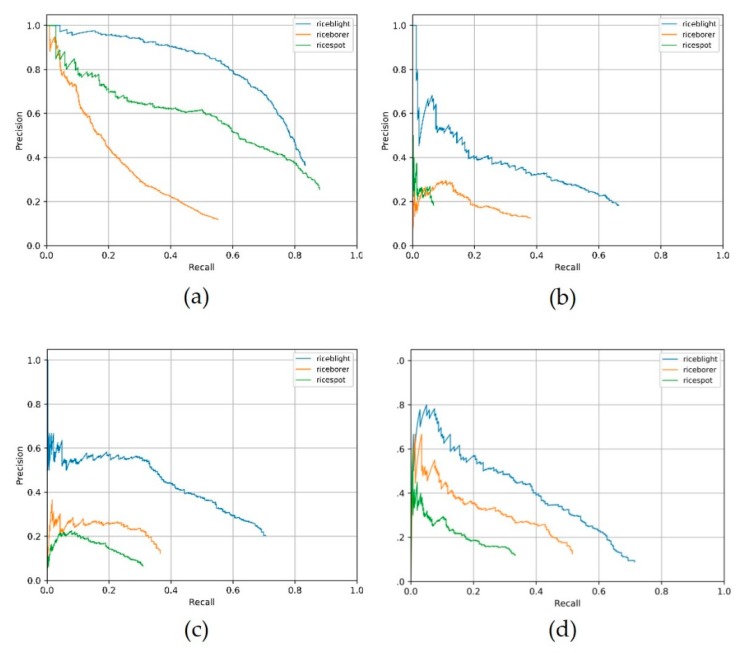
Precision-Recall curve of the still-image detector of the three other backbones and our custom DCNN backbone. Blue, yellow and green line denotes rice sheath blight, rice stem borer symptoms and rice brown spot respectively. (**a**) VGG16 with pre-trained model, (**b**) ResNet-50 with pre-trained model, (**c**) ResNet-101 with pre-trained model, (**d**) the custom DCNN training from scratch.

We trained these four backbones with pre-trained models using the platform Detectron, the metrics are shown in Table 7. Our DCNN used VGG16 as the pre-trained model, since the custom DCNN’s architecture was similar to VGG16. The video metrics of these four backbones from scratch (training with no pre-trained model) [52] are shown in Table 8. “Custom DCNN from scratch” in Table 8 is the backbone illustrated in Section 2.3.3, which we adopt ultimately. The symbol of “/” denotes the situation of zero divided by zero. Precision-Recall (PR) curve of the four contrastive backbones in the still-image detector is shown in Figure 9. We selected the four best effect PR curve images, which were vgg16 with pre-trained model, ResNet-50 with pre-trained model, ResNet-101 with pre-trained model and the proposed DCNN which was trained from scratch. Note that the PR curves are of the still- image detectors, but not of the video detection system, and the test-set which generates the PR curve is the clearer images, not the relatively blurrier frames, thus the backbone of better PR curve might not produce better video detection results in our experiments.

The rice brown spot video precision of VGG16 and rice stem borer video precision of the custom DCNN are 100% in Table 7. Video precision of 100% means the boxed lesion spots in Video 1 are all correct in each category. VGG16, ResNet-50 and ResNet-101′s performance on rice stem borer symptoms were not well. We acknowledge the results are of our experimental environment, but we repeated our experiments using Video 1 on different Linux servers and confirmed this result. We used another server with Ubuntu 18.04, CUDA 9.0 and the 1080ti GPU, the experimental results were nearly the same as the above results in Table 7 and Table 8. We assume that the reason why the three backbone has a non-ideal result on rice stem borer symptoms is the non-specific symptoms of rice stem borer. Figure 1 shows that the withered leaves of rice stem borer symptoms are similar to those of rice sheath blight, but there are still some minor differences between them. The withered stems and leaves in Figure 1a appear to have plaques, while those in Figure 1b appear not to have. Our backbone showed better sensitivity (video recall in our experiments) in extracting subordinate features (e.g., such as the fungal plaques). Our experiments also showed that training from scratch might have an advantage over training with a pre-trained model, especially in rice detection.

Knowledge transfer (using pre-trained neural network models) generally reduces training time. In our experiments, the training time of ResNet-50 with a pre-trained model was nearly one hour less than that of training ResNet-50 from scratch. But from the results of Table 7 and Table 8, the metrics of ResNet-50 with a pre-trained model were worse than that of training ResNet-50 from scratch. We assume the reason may be that we use the image-training model to detect relatively blurry videos.

We assume the reason why VGG16, ResNet-50, and ResNet-101 performed not so well in our experiments is that video frames are not always as clear as photos. We used the model trained with clear images to detect blurry video frames. Though what we used were the HD videos, the video frames were still not as clear as photos because of the video defocus and motion blur. The features between clear images and blurry frames might be much different. Thus, those backbones which are special for images may not be suitable for the detection of less clear frames.

For another reason, pre-trained models might have the possibility to disadvantageously affect training results. Since the pre-trained model was trained from other objects, the features of other objects might be quite different from the crop lesions. This might be a finding, i.e., for some special-purpose recognition, the detection rate without a pre-trained model might be higher than that with a pre-trained model.

### 4.6. Comparison with the State-of-the-Art Method YOLOv3

**Figure 10:**
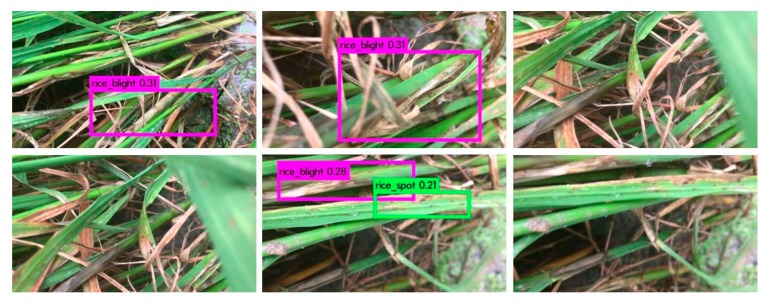
Detection results of YOLOv3 using Video 1. The red box denotes rice sheath blight, green box denotes rice brown spot. Video 1 was captured in Hunan Province, China, July 2018, which was illustrated in Figure 6. The detection results of YOLOv3 was not good as the custom DCNN system from the comparison of the figure and Figure 6.

**Figure 11:**
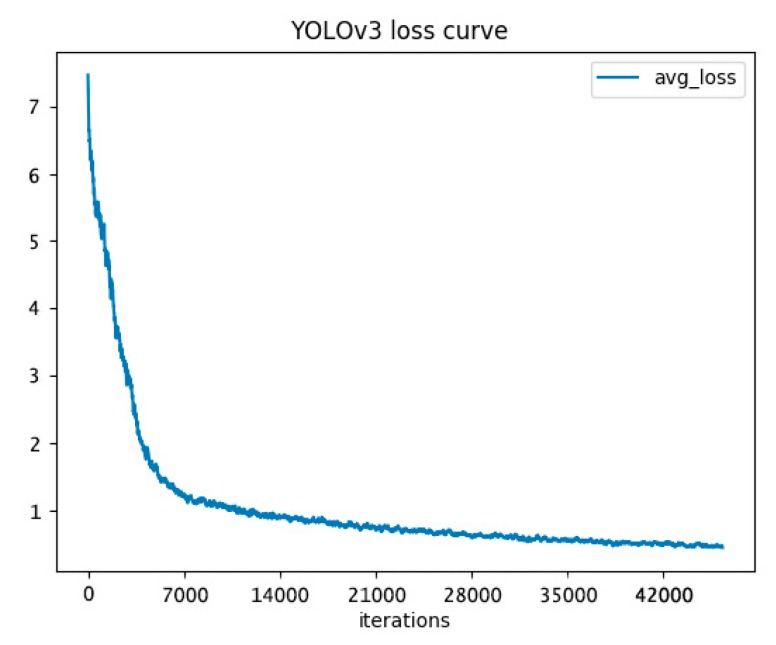
Loss curve of YOLOv3. We set the iterations to 50,000. Other parameters are described in Section 3.1. The training was stopped when achieving the max iterations.

YOLOv3 [22] is a state-of-the-art method of real-time video object detection. We applied it to rice diseases and pests video detection. We chose the hyperparameters after experiments. The hyperparameters were illustrated in Section 3.1. We used Video 1 in the contrastive experiment. The model with 30,000 iterations achieved the best detection effect with our experimental environment. The video captures are visualized in Figure 10, the loss curve of YOLOv3 is visualized in Figure 11, and the video metrics are listed in Table 9.

YOLOv3 had a good detection effect on the spots with clear boundaries and regular shapes, especially for the shapes that were similar to the training images, but had a non-ideal detection effect on spots with blurry boundaries and irregular shapes.

The sensitivity (video recall) of our video detection system was higher than YOLOv3 in Table 9, while YOLOv3 had very high detection precision (video precision). Low video recall denotes that a few of correct lesion spots are boxed out in our experimental environment, video precision of 100% denotes that the boxed lesion spots are all correct. YOLOv3 has a real-time detection speed, our experiments showed that the recall was still lower than the two-stage system in rice diseases and pests detection. Additionally, as YOLOv3 divides pictures into grid cells for prediction, the bounding box’s location may not be as accurate as our Faster-RCNN based detection system. But most of all, we did not achieve the video detection speed of YOLO, which was the purpose of our building the system.

We assume that the reasons of the low video recall of YOLO are as follows: (a) YOLO reduces the size of the input image to 608 × 608 pixels (in our experiments), which may reduce the effective pixels; (b) the two-stage system which has the specific feature extractor, may be appropriate for the rice diseases and pests video detection under complex background; (c) there may be the video detection difficulties for YOLO on rice diseases and pests illustrated in Section 4.8.

### 4.7. Supplementary Evaluation of Our System

The custom DCNN backbone had eminent detection sensitivity (video recall) in withered leaves of rice sheath blight and rice stem borer symptoms. It might distinguish different withered leaves in our experimental environment, e.g., withered leaves of rice sheath blight might have fungal infection, while withered leaves of rice stem borer symptoms might have none (Figure 1). From Figure 1a, it could be seen that withered leaves were a feature of rice sheath blight. The custom DCNN video detection results of withered leaves are shown in Figure 6 and Figure 8. The other backbones in the experiments might not have good sensitivity of withered leaves in Table 7 and Table 8, as the withered leaves of rice stem borer were not detected by the other three backbones in Table 7. In Table 9, YOLOv3′s video precision was high, which denoted the detected spots were all correct, but the video recall was low, which denoted the detection rate of true lesions was low. As shown in Figure 4, we did not annotate the withered leaves of rice sheath blight, but they could be detected by the custom DCNN system in our experiment. It might denote the custom DCNN backbone had high sensitivity in detecting subordinate features, such as the withered leaves.

### 4.8. Difficulties in Rice Video Detection

Firstly, like other video detection, rice video detection also has problems such as video defocus, motion blur and part occlusion. These may lead to misdetection, or low detection confidence. Secondly, the shape of lesion spots is always irregular, and the boundaries of lesions are always non-obvious. Meanwhile, because of the existence of video defocus and motion blur, the lesion spots of each frame appear fuzzier.

Thirdly, we used the relatively clearer images (as shown in Figure 3) to train the model, and used the model to detect relatively blurry frames. Additionally, the situations of the frames were more complex, such as the disordered distribution of rice plants, the mixture of plants, water and mud, and the mixture of several lesions (as shown in Figure 2). Therefore, the extracted features from the video frames would be different from the trained features, which might lead to misdetection.

### 4.9. Shortcomings of Our System

Firstly, our video detection system had a relatively long video detection time. Since we used HD videos for detection and detected every frame, the detection time of the 15s 1080P video was about 40s to 50s. Secondly, we did not extract the key frames, while the information of the adjacent frames was mostly repeated, which resulted in the waste of computing resources and detection time. Thirdly, our detection confidence was relatively low. In the case of video defocus, motion blur, the irregular shape and non-obvious bounding of the lesion spots, our detection confidence was not high, and our detection threshold of confidence was set low, too. Finally, the metrics were calculated manually. To compute them automatically is a difficulty. These are all our next research directions.

## 5. Conclusions

In this paper, we accomplished three categories of rice diseases and pests video detection, which were rice sheath blight, rice brown spot, and rice stem borer symptoms. The video detection way was that we used image-training models to detect untrained videos. We proposed a set of video detection metrics, which was consistent with the detected video in our experimental environment. In addition, we designed a custom DCNN backbone network for video detection, which was suitable for rice video detection with our experimental environment and our detection way. We found that VGG16, ResNet-50 and ResNet-101 might have an unsatisfactory detection effect on slightly blurry images. Furthermore, we tested the state-of-the-art method YOLOv3 on rice lesion spots with the same detection threshold of confidence, and found that YOLOv3 had a non-ideal detection result to objects with blurry boundaries and irregular shapes. We consider that our system could be applied to other crop diseases and pests.

## Figures and Tables

**Table 1:** Statistics of the images in the dataset collected in China, 2018. The images were used for training and testing the still-image models.

Symptom	No. of Images	No. for Training	No. for Test
Rice sheath blight	1800	1620	180
Rice stem borer symptoms	1760	1584	176
Rice brown spot	1760	1584	176
Total	5320	4788	532

**Table 2:** Statistics of the videos in the dataset collected in China, 2018. The videos were only for detection. They did not participate in the model training.

Video Name	Main Symptom	Frames
Video 1	Rice sheath blight	450
Video 2	Rice stem borer symptoms	1650
Video 3	Rice brown spot	2190
Video 4	Rice sheath blight	390
Video 5	Rice stem borer symptoms	2220
Total		4290

**Table 3:** The custom DCNN architecture.

	Layer	Kernel Size	Stride	Repeat	Output Channels
	Image				3
block 1	Conv. 1	3 × 3	1	3	64
	ReLU				
	LRN				
	MaxPool	2 × 2	2		
block 2	Conv. 2	3 × 3	1	3	128
	ReLU				
	LRN				
	MaxPool	2 × 2	2		
block 3	Conv. 3	3 × 3	1	3	256
	ReLU				
	LRN				
	MaxPool	2 × 2	2		
block 4	Conv. 4	3 × 3	1	3	512
	ReLU				
	LRN				
	MaxPool	2 × 2	2		

**Table 4:** Lesion statistics and metrics of Videos 1–3 using the custom DCNN system ^1^. The videos are illustrated in Section 2.1.

	Video 1	Video 2	Video 3
No. of true spots	50	67	38
No. of spots detected	39	91	40
No. of true spots detected	34	60	28
Video recall	68.0%	89.6%	73.7%
Video precision	87.2%	65.9%	70.0%
F1 score	76.4	75.9	71.8

^1^ The calculation of them is to use Equations (1)–(3), which are illustrated in Section 2.2. The spots include the three categories of rice diseases and pests, which are rice sheath blight, rice stem borer symptoms, and rice brown spot. The metrics are the total statistic of the three categories. Videos 1–3 were not included in the training and test dataset, they were only for detection.

**Table 5:** Video metrics of Video 4 and Video 5 with the custom DCNN system (%). Video 4 and Video 5 were used for validating the system further.

Video Metrics	Blight Recall	Blight Precision	Borer Recall	Borer Precision	Spot Recall	Spot Precision
Video 4	78.6	84.6	66.7	50.0	75.0	85.7
Video 5	66.7	50.0	83.3	76.9	70.0	63.6

**Table 6:** Video metrics of different depth of the custom DCNN backbone using Video 1 (%). Video 1 is illuminated in Section 2.1.

Video Metrics	Blight Recall	Blight Precision	Borer Recall	Borer Precision	Spot Recall	Spot Precision
14 conv. layers	74.1	64.5	9.1	3.8	8.3	5.3
11 conv. layers	29.6	72.7	18.2	15.4	33.3	40.0
Custom DCNN	74.1	90.9	45.5	71.4	75.0	90.0

**Table 7:** Video metrics of backbones with pre-trained models using Video 1 (%) ^1^. Video 1 is illuminated in Section 2.1.

Backbone	Blight Recall	Blight Precision	Borer Recall	Borer Precision	Spot Recall	Spot Precision
VGG16	51.9	100.0	0	/	8.3	100.0
ResNet-50	55.6	93.8	0	/	0	/
ResNet-101	59.3	100.0	0	/	41.7	60.0
Custom DCNN	70.4	90.5	9.1	100.0	0	0

^1^ In Video 1, the total number of true spots of the three categories was 50, the total number of spots detected was 39, the total number of true spots detected was 34. The numbers were counted manually. The same spot in a video was not repeatedly counted. Video 1 was captured in Hunan Province, China, July 2018. VGG16, ResNet-50, ResNet-101 failed to detect rice stem borer. The symbol “/” is used to denote zero divided by zero.

**Table 8:** Video metrics results of backbones from scratch using Video 1 (%) ^1^.

Backbone	Blight Recall	Blight Precision	Borer Recall	Borer Precision	Spot Recall	Spot Precision
VGG16	18.5	100.0	9.1	8.3	16.7	8.7
ResNet-50	59.3	94.1	45.5	83.3	66.7	88.9
ResNet-101	66.7	94.7	0	/	16.7	40.0
Custom DCNN	74.1	90.9	45.5	71.4	75.0	90.0

^1^ The parameters of the video are the same as the video in Table 7. ResNet-101 failed to detect rice stem borer when training with no pre-trained model in our experiments.

**Table 9:** Video metrics of YOLOv3 and our system using Video 1 (%) ^1^.

Video Metrics	Blight Recall	Blight Precision	Borer Recall	Borer Precision	Spot Recall	Spot Precision
YOLOv3	29.6	100.0	9.1	100.0	25.0	100.0
Custom DCNN System	74.1	90.9	45.5	71.4	75.0	90.0

^1^ Video 1 is the video used in Figure 6 and Figure 10, which was captured in Hunan Province, China, July 2018. YOLOv3 had a high precision, but had a low recall in our experiment.

## Supplementary Materials

The following are available online at https://www.mdpi.com/1424-8220/20/3/578/s1, including all the figures and tables in the paper, architecture algorithm of the rice video detection, and the detected videos.
