# Supplementary material for: A Recognition Method for Rice Plant Diseases and Pests Video Detection Based on Deep Convolutional Neural Network
Source: Sensors (Basel). 2020 Jan 21;20(3):578. doi: 10.3390/s20030578 (PMC7038217; doi:10.3390/s20030578)
Supplement: Supplementary file 1 [file sensors-20-00578-s001.zip › sensors-663740-supplementary/Architecture algorithm of the rice disease video detection.docx]

| **Algorithm 1** Architecture algorithm of the rice disease video detection.  1:training a still-image model in the Still-image Object Detector  2:**input**: a video |
| --- |
| 3:n_frame ← 0 ▷ n_frame: number of frame that has been detected  4:cap ← the video ▷ by using OpenCV |
| 5:**while** true **do**  6: n_frame ← n_frame + 1  7: read a chronological frame from cap ▷ by using OpenCV |
| 8: Transmit the frame to the Still-image Object Detector to detect with the model, generate detection boxes and confidence in the frame  9: img ← the detected frame |
| 10: Synthesize video from img ▷ by using OpenCV |
| 11: **if** the next frame is vacant **then**  12: **break** |
| 13:**end while**  14:**output**: a detected video with classes and confidence  15: n_frame ▷ the number of the frames of the video |
